# The impact of malocclusions on oral health-related quality of life in children—a systematic review and meta-analysis

**DOI:** 10.1007/s00784-015-1681-3

**Published:** 2015-12-04

**Authors:** Lea Kragt, Brunilda Dhamo, Eppo B. Wolvius, Edwin M. Ongkosuwito

**Affiliations:** 1 Department of Oral & Maxillofacial Surgery, Special Dental Care and Orthodontics|, Erasmus Medical Center, Rotterdam, The Netherlands; 2 Department of The Generation R Study Group, Erasmus University Medical Center, Rotterdam, The Netherlands; 3 P.O Box 2040, 3000CA Rotterdam, The Netherlands

**Keywords:** Meta- analysis, Quality of life, Malocclusions, Children

## Abstract

**Introduction:**

A limited amount of systematic literature reviews on the association between malocclusions and oral health-related quality of life (OHRQOL) summarize inconclusive results. Therefore, we conduct a systematic review and meta-analysis on the association of malocclusions with OHRQOL in children.

**Methods:**

Relevant studies were identified in Pubmed, Embase, Cochrane, Google Scholar and other databases. All studies with data on malocclusions or orthodontic treatment need and OHRQOL in children were included. Methodological quality of the studies was assessed with the Newcastle-Ottawa Scale (NOS). Random effects models were used to estimate summary effect measures for the association between malocclusion and OHRQOL in a continuous and a categorical data analysis. Tests for heterogeneity, publication bias and sensitivity of results were performed.

**Results:**

In total, 40 cross-sectional studies were included in the meta-analyses. Summary measures of the continuous data show that OHRQOL was significantly lowered in children with malocclusions (standardized mean difference (95 % CI] = 0.29 (0.19–0.38)). The summary odds ratio for having an impact on OHRQOL was 1.74 times higher in children with malocclusion than in children without malocclusions. Heterogeneity among studies was partly explained by malocclusion assessment, age of the children and country of study conduction.

**Conclusion:**

Our results provide evidence for a clear inverse association of malocclusion with OHRQOL. We also showed that the strength of the association differed depending on the age of the children and their cultural environment.

**Clinical relevance:**

Dentists benefit from understanding the patient differences regarding the impact of malocclusions.

**Electronic supplementary material:**

The online version of this article (doi:10.1007/s00784-015-1681-3) contains supplementary material, which is available to authorized users.

## Introduction

Malocclusion is one of the most common oral disorders in the Netherlands. In 2005, half of the Dutch adolescents have had orthodontic treatment, and in 2011, this proportion increased to 60 % [1]. A variety of deviant occlusal traits exist that in itself can vary in severity.

The concept of oral health-related quality of life (OHRQOL) arose in the orthodontic literature to explain the variability in professionally determined (objectively) and patient-determined (subjectively) need for orthodontic treatment [2, 3]. OHRQOL is a patient-reported outcome assessed by questionnaires to measure the psychological impact of the dentition. More precisely, OHRQOL is the interplay of oral health variables such as biological and physiological functional status, as well as personal attributes like role functioning, social functioning and psychological functioning, that represent the multidimensional and individual perception of oral health [4]. In this way, it describes the standard of the oral and related tissues which enables an individual to eat, speak and socialize without active disease, discomfort or embarrassment and which contributes to general well-being [5]. In the last 15 years, the literature on the association of malocclusions and OHRQOL has greatly expanded [6].

Most studies in the orthodontic literature on OHRQOL use small convenience samples, which limits their evidence. In 2006, Zhang et al emphasized the impact of heterogeneous population groups and measurement tools on the conflicting evidence in orthodontic OHRQOL research [3]. Indeed, Liu et al reviewed the literature in 2009, but found only a modest association between malocclusion and the quality of life among mixed ages [2]. A recent meta-analysis on malocclusions, orthodontic treatment and OHRQOL in adults found a moderate increase of OHRQOL after treatment (standardized mean difference (SMD)(95 % CI) 1.29 (0.67–1.92)), but the difference in OHRQOL between people with and people without malocclusion was small (SMD (95 % CI) 0.84 (0.25–1.43)) [7]. Both reviews suffered from the considerable differences in study design.

The impact of malocclusions and OHRQOL might be different in children than in adults as they deal differently with disease, but also with psychological, social and emotional factors [8]. In addition, children and adult OHRQOL measures are different; thus, they should not be investigated simultaneously. When the focus lies on OHRQOL in children, a variety of instruments exist without one universally accepted. One of the first instruments used in adolescents is the Oral Health Impact Profile-14 (OHIP-14) [9]. In 2002, the Child Perception Questionnaire (CPQ) was developed and further developed into the Child Oral Health Impact Profile (COHIP) [8, 10]. And in 2004, the Child Oral Impact of Daily Performances (OIDP) was derived from its adult form [11]. Finally in 2007, an instrument for very young children was developed, the Early Childhood Oral Health Impact Scale (ECOHIS) [12]. These various instruments have much in common, but there are also differences as some focus on the severity whereas others focus on the frequency of oral impacts on OHRQOL, or some instruments make use of parent forms whereas other address the questions directly to the children. A systematic review and meta-analysis with a sufficient amount of studies could explore and explain the influence of differences among studies on the association between malocclusion and OHRQOL. Because the majority of orthodontic patients are children and adolescents, this review focusses on the relationship of malocclusions or orthodontic treatment need and OHRQOL in subjects up to 18 years old.

## Aim of the study

The primary objective of this study is to give a complete overview on the influence of malocclusion, assessed as occlusal trait or orthodontic treatment need, on OHRQOL measured with validated questionnaires in children and adolescents.

The secondary objective of this study is to explain the differences in the association between malocclusion and OHRQOL in children by investigating the sources of heterogeneity among the included studies.

## Material and methods

The present study was performed according to the guidelines of the PRISMA statement for conducting a systematic review [13]. The review protocol can be accessed via the webpage: http://www.crd.york.ac.uk/PROSPERO/ (Registration number: CRD42015019522).

### Literature search

Relevant articles about the impact of malocclusion and orthodontic treatment need on OHRQOL measured by a questionnaire were retrieved by searching Medline OvidSP, Embase, Web-of-sciences, Cochrane central, PsycINFO, OvidSP, Scopus, PsycINFO, Cinahl and finally Google Scholar. The search strategy was built with text words and medical subject headings (MeSH). The main terms were orthodontics, (different) malocclusions, treatment need, quality of life and self-perception. The term self-perception was added to the search strategy to ensure that all articles were found with outcome on OHRQOL. The full search strategy was built with the support of the librarian of the Erasmus Medical Center and is available in the supplemental material (S1). The search was performed by two reviewers (LK, BD) independently. At first, the titles of all articles were screened for their relevance. Here upon, the abstracts of relevant articles were retrieved and read. After the abstract selection, full-text copies of the selected papers were retrieved and the final selection for inclusion was made. After both reviewers performed the complete selection procedure, the results of the searches were compared and discussed in case of disagreement.

### Study selection

For this systemic review all original and peer-reviewed human studies on the relationship of orthodontic treatment need or malocclusion with OHRQOL in children were searched. The first search was conducted to include all articles until June 2013; a second search was performed to update the relevant articles in September 2014. Finally, the search in PUBMED was repeated in September 2015 to check whether new relevant articles were available. For the selection of studies, predefined criteria were used.

All English-written studies providing quantitative information about the association of malocclusions with OHRQOL assessed by a questionnaire validated for the use in children were included.

Letters to the editors, conference proceedings, unpublished studies, case reports and series as well as reviews were excluded from the study selection. When multiple papers were identified on the same population, the study with more information on the data was included in the present review. Studies with participants requiring orthognathic surgery or with syndromic patients were excluded. Also, studies using general (health related) quality of life measures were excluded. Studies that only measured the impact of orthodontic treatment or had a before-after design were excluded when they had no appropriate information on control groups before treatment started. Also, studies with children that already had orthodontic treatment or studies that did not use a healthy comparison group (no or less malocclusion resp. orthodontic treatment need) were excluded from this review. Finally, only studies with subjects having a mean age under 18 years were included in this review.

**Fig. 1:**
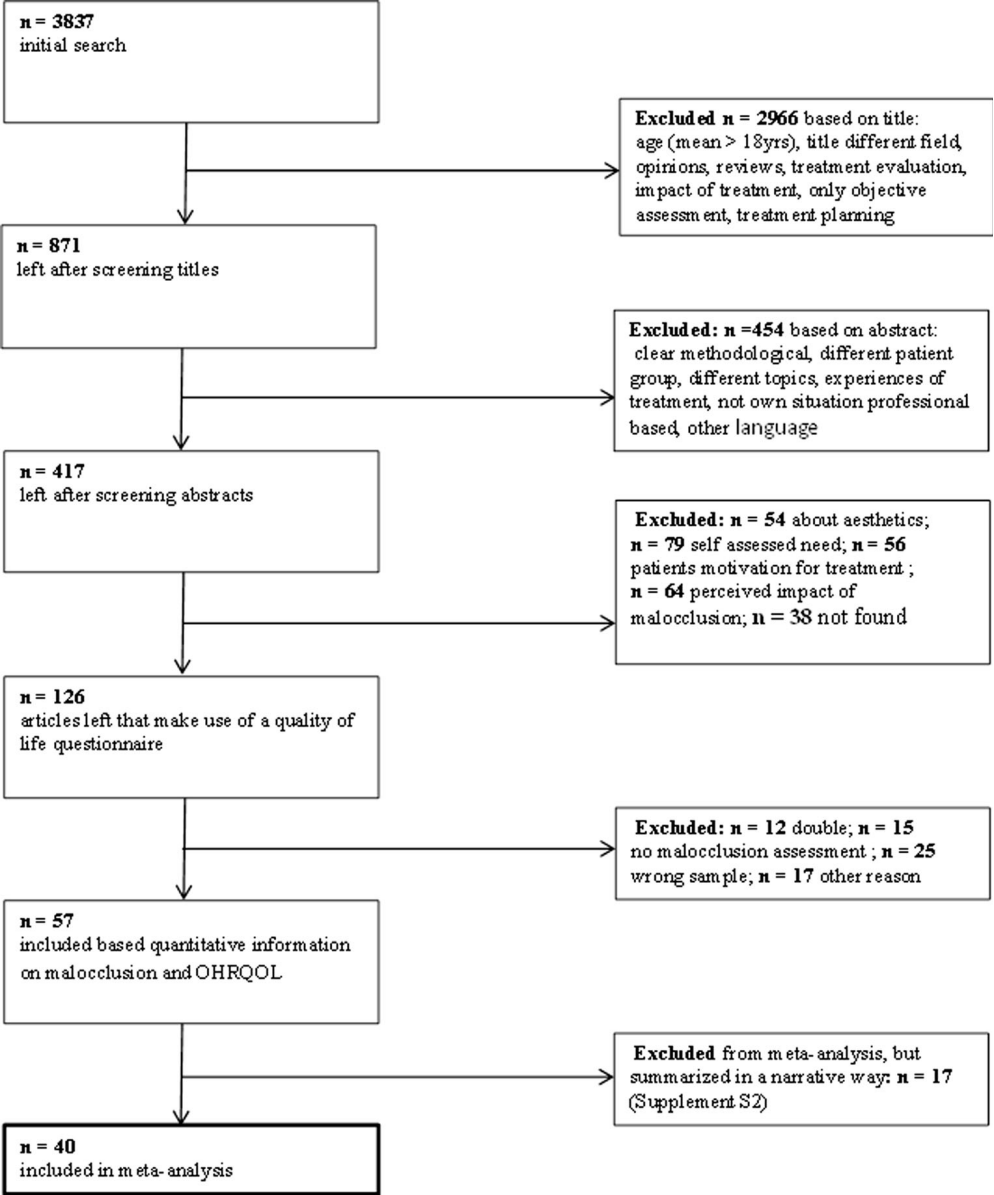
Flowchart of study selection

Studies that did not provide sufficient information on the number of participants and number of patients with impacts on OHRQOL or means with standard deviation of OHRQOL per subgroup, either directly or to be calculated, were excluded from the meta-analysis, but summarized in a narrative way. Studies that assessed orthodontic treatment need only with the aesthetic component of the index of orthodontic treatment need (IOTN-AC) were also excluded from the meta-analysis, but included in the narrative review, because it is not clear whether the IOTN-AC is assessed by the professional or the patient. In Fig. 1, the flowchart of the study selection is presented. The narrative review is available in the supplement (supplement S2).

### Data extraction

From the final set of relevant studies, the following data were extracted: study characteristics (first author, publication year, country where the study was conducted, study design, study size, number of cases and controls), the description and assessment of the exposure (malocclusion or orthodontic treatment need) and description of the outcome assessment (name, length and administration of the questionnaire) (Table 1). For studies that used a continuous OHRQOL measure, the mean and standard deviations of OHRQOL as well as the number of subjects per subgroups were extracted. For studies that used a dichotomous OHRQOL measure, the number of patients with lowered OHRQOL per subgroup as well as the total number of subjects per subgroup was extracted. If a study reported on more than one occlusal index or OHRQOL measure, results from all were extracted to be used for subgroup analysis. For the overall summary measure, the results based on the Dental Aesthetic Index (DAI) were used [14–17]. One study reported results with two OHRQOL measures, in which the only difference was the specific age group, i.e. CPQ8-10 and CPQ11-14 [18]. For this study, both results are included in the meta-analysis. In three studies, a generic and a condition-specific OHRQOL measure was used, but for the analyses, the condition-specific measure was taken only [14, 19, 20].

**Table 1:** Study characteristics of the studies included in the narrative review and meta-analysis (*N* = 40)

	Author	Year	Country	Study type	Participants	Age (mean)	*N*	OHRQOL	Malocclusion	Ref
1	Abanto, J.et al.	2011	Brazil	Cross-sectional	Preschool children seeking dental care	3.8	260	ECOHIS	Anterior trait	[52]
2	Aguilar-Diaz, F.C. et al.	2011	Mexico	Cross-sectional	Children living in San Luis Potosi	8.98	212	CPQ8-10	DAI	[23]
3	Anosike, A. N. et al.	2010	Nigeria	Cross-sectional	Children randomly selected from secondary schools	14.5	805	OHIP-14	DAI	[57]
4	Asgari, I. et al.	2013	Iran	Cross-sectional	High school children	14.9	597	COHIP	IOTN	[35]
5	Barbosa, T.S. et al.	2009	Brazil	Cross-sectional	School children	9.0/12.5	90 /120	CPQ8-10/11-14	DAI	[18]
6	Bhayat, A. et al.	2014	Saudi Arabia	Cross-sectional	Male school children	–	268	CPQ11-14	Angle’s classification	[43]
7	Bekes, K. et al.	2012	Germany	Cross-sectional	Recruited from annual screening	12.3	1061	CPQ11-14	Presence/absence	[42]
8	Bernabe, E. et al.	2008	UK	Cross-sectional	School children	16.42	200	CS-OIDP/OHIP-14	DAI/IOTN	[14]
9	Bernabe, E. et al. (1)	2008	Brazil	Cross-sectional	Students from secondary schools	–	220	CS-OIDP	Angles classification	[47]
10	Bernabe, E. et al. (1)	2009	Brazil	Cross-sectional	Students from secondary schools	–	1060	OIDP/ CS-OIDP	DAI	[19]
11	Bernabe, E. et al.	2009	Thailand	Cross-sectional	School children		1034	OIDP/CS-OIDP	IOTN	[20]
12	Brown A. et al.	2006	Saudi Arabia	Cross-sectional	Dental patients	–	174	CPQ11-14	None/slight/moderate/severe (IOTN)	[33]
13	Carvalho, A. C. et al.	2013	Brazil	Cross-sectional	Preschool children and daycare children	5.4	1069	ECOHIS	Presence/Absence	[53]
14	Dawoodbhoy, I. et al.	2013	Saudi Arabia	Cross-sectional	Hospital volunteers	12.6	278	CPQ11-14	DAI	[24]
15	Feu D. et al.	2010	Brazil	Cross-sectional	Prospective patients and school children	13.6	194	OHIP-14	IOTN-DHC/AC-IOTN	[58]
16	Foster Page, L.A. et al.	2005	New Zealand	Cross-sectional	School children	12.7	430	CPQ11-14	DAI	[25]
17	Foster Page, L.A. et al.	2013	New Zealand	Cross-sectional	Dunedin adolescents		353	CPQ11-14	DAI	[26]
18	Ghijselings, I. et al.	2014	Belgium	Cross-sectional	Prospective orthodontic patients	13.8	386	CPQ11-14/OASIS	IOTN-DHC/IOTN-AC	[44]
19	Gomes, M. C. et al.	2014	Brazil	Cross-sectional	Preschool children	3.95	843	ECOHIS	Presence/absence	[54]
20	Herkrath, F. J. et al.	2012	Brazil	Cross-sectional	School children	12	201	C-OIDP	IOTN-DHC, DAI	[48]
21	Hvaring, C. L. et al.	2014	Norway	cross-sectional	Patients	13.6	163	OIDP	Hypodontia	[49]
22	Kolawole, et al.	2011	Nigeria	cross-sectional	School children	12.54	248	CPQ11-14	DAI	[27]
23	Kragt, et al.	2015	The Netherlands	Cross-sectional	Referred to orthodontic clinic	11.85	243	COHIP	IOTN	[36]
24	Kramer, P. F. et al.	2013	Brazil	Cross-sectional	School children	3.5	1036	ECOHIS	Presence/absence	[55]
25	Laing, E. et al.	2010	UK	Cross-sectional	Prospective orthodontic patients	13.6	123	CPQ11-14	Hypodontia	[45]
26	Locker, D. et al.	2007	Canada	Cross-sectional	Prospective orthodontic patients	12.5	141	CPQ11-14	DAI/PAR	[28]
27	Manijth, C. M. et al.	2012	India	Cross-sectional	Adolescents seeking orthodontic treatment	13.0	200	OHIP-14	IOTN-DHC	[22]
28	Marques, L. S. et al.	2006	Brazil	Cross-sectional	School children	13.2	333	OIDP	DAI/any malocclusion	[15]
29	Mbawalla, H. S. et al.	2011	Tanzania	Cross-sectional	School children from two sides	13.0	2678	CS-OIDP	Presence/absence	[50]
30	Montiel-Company, J. M. et al.	2013	Spain	Cross-sectional	School children	13.25	627	PIDAQ	IOTN/DAI	[16]
31	Onyeaso, et al.	2009	Nigeria	Cross-sectional	School children	–	274	OHIP-14	ICON	[34]
32	Paula, J. S. et al.	2012	Brazil	Cross-sectional	School children	–	515	CPQ11-14	DAI	[46]
33	Paula Jr, et al.	2011	Brazil	Cross-sectional	Adolescents from public high school	16.1	301	PIDAQ	DAI	[32]
34	Peres, K. G. et al.	2013	Brazil	Cross-sectional	Brazilian Oral Health Survey (SB Brazil 2010)	16.84	5445	OIDP	DAI	[51]
35	Sardenberg F. et al.	2013	Brazil	Cross-sectional	School children	–	1204	CPQ8-10	Presence/absence	[41]
36	Scapini, A. et al.	2013	Brazil	Cross-sectional	School children	–	632	CPQ11-14 /ISF:20	DAI	[29]
37	Scarpelli, A. C. et al.	2013	Brazil	Cross-sectional	Preschool children	–	1632	ECOHIS	Presence/absence	[56]
38	Schuch, H. S. et al.	2014	Brazil	Cross-sectional	School children	9.15	750	CPQ8-10	DAI	[30]
39	Ukra, A et al.	2013	New Zealand	Cross-sectional	School children	–	783	CPQ11-14	DAI	[31]
40	Zang, M. et al.	2009	China	Cross-sectional	Sample seeking orthodontic treatment	13.2	121	CPQ11-14	DAI/ICON/IOTN	[17]

### Data synthesis

When data were presented separately for girls and boys, these were combined to one group. Mean and standard deviations were re-calculated following the Cochrane Handbook [21]. One study did not present results on the overall OHRQOL but presented the result per questions [22]. In this case, the OR (95 % CI) was calculated per question. Afterwards, all OR (95 % CI) were pooled with a fixed effects meta-analysis, and the number of events per subgroup were re-calculated proportional to the sample size of the study.

All analysis were performed with a dichotomous independent variable malocclusion (malocclusion vs no malocclusion). Therefore, for the studies that presented their results in more defined subgroups, e.g. a borderline need category, the subgroups were re-grouped following the guidelines of the Cochrane Handbook [21]. The following cut-offs for orthodontic treatment need indices were used to indicate no malocclusion: For the *Dental Aesthetic Index* (*DAI*), the value of ‘minor/none’, grade one or a score ≤25 was used [14, 16–19, 23–32], for the *IOTN*, the grade ≤3 or borderline need was used [14, 17, 20, 33] and for the *Index of complexity outcome and need* (*ICON*), a score ≤31 or a cut-off value of ≤43 was used [17, 34].

OHRQOL was assessed with various questionnaires among the different studies. In general, all measures indicated better OHRQOL with a lower score. Only the *COHIP* indicated better OHRQOL with a higher score [35, 36]. In the meta-analysis, the absolute mean differences were used for the results based on the COHIP to make them comparable to the results of other studies in the meta-analysis.

### Quality assessment

We assessed the methodological quality of the individual studies with the Newcastle-Ottawa Scale (NOS) adapted for cross-sectional studies [37, 38]. This scale rates the quality of the included studies on three topics: selection of the study population, comparability of the groups under study and the outcome assessment. The maximum score of this scale is 10, and we assigned high methodological quality to a study if a score >5 was given.

### Statistical analysis

Statistical analyses were performed in Review Manager 5.3 from the Cochrane Collaboration.

The studies were analysed in two ways. On the one hand, studies that used a continuous OHRQOL scale (mean ± SD) were grouped in one meta-analysis. On the other hand, studies that used a categorical OHRQOL outcome (no impact vs impact ) were grouped into another meta-analysis. This grouping was not mutually exclusive and when possible, we included the studies in both meta-analyses.

Random effect models were used for the meta-analyses to calculate summary SMD with 95 % CIs for the continuous analyses and summary OR (SOR) with 95 % CIs for the categorical analysis.

Heterogeneity was assessed with the *I* ^2^-statistic. The *I* ^2^-statistic quantifies the relative inconsistency between studies. *I* ^2^ values above 50 % were considered to indicate substantial inconsistency due to heterogeneity [39]. First, studies were grouped based on their outcome measure, i.e. the OHRQOL questionnaire. After that, we stratified the analysis where possible by the following predefined variables to explain heterogeneity and inconsistency in results: Malocclusion assessment, mean age of the study population, country of study conduction and whether the sample was recruited from schoolchildren or from prospective orthodontic patients. Studies using the CPQ as OHRQOL measure were stratified on age-specific measurements instead of mean age. We tested for subgroup differences with the chi-square test.

Small study bias, respectively publication bias, was inspected in funnel plots [40]. An asymmetric funnel shape was used to inspect a biased relationship between study size and effect size. We performed sensitivity analyses to test the robustness of the summary estimate by omitting one study at a time from the random effects model. We also tested for differences in summary estimates between high- and low-quality studies for both meta-analysis.

## Results

### Malocclusion assessment and OHRQOL measures of studies included in meta-analysis

The most commonly (*n* = 18) used OHRQOL questionnaires were the two CPQs, i.e. for the age group 8–10 years [18, 23, 30, 41] and the age group 11–14 years [17, 18, 24–29, 31, 33, 42–46]. Also, the OIDP was often (*n* = 9) used in children and adolescents of 10–19 years old [14, 15, 19, 20, 47–51]. Five studies used the ECOHIS in 1–5-year-old children [52–56]. Two studies used the COHIP [35, 36] in children from 9–18 years and five studies used the OHIP-14 in children aged 11–17 years [14, 22, 34, 57, 58]. Finally, two studies used the Psychosocial Impact of Dental Aesthetics Questionnaire (PIDAQ) to measure OHRQOL in 12–20-year-old children/adolescents [16, 32]. One study used additionally the Oral Aesthetic Subjective Impact Scale (OASIS); however, this questionnaire is not further considered in this review [44].

Studies used several methods to assess malocclusions or orthodontic treatment need in their study population. Most of the time (*n* = 19), the DAI was used [14–19, 23–32, 46, 51, 57]. The IOTN-Dental Health Component (DHC) was used in 11 studies [14, 16, 17, 20, 22, 33, 35, 36, 44, 48, 58]. The ICON was used in two studies [17, 34], and Angle’s classification system was also used in two studies [43, 47]. Two studies assessed the relationship of tooth agenesis and OHRQOL [45, 49]. Finally, nine studies assessed the presence of any malocclusion trait or anterior malocclusion trait [15, 41, 42, 50, 52–56].

### Meta-analysis

**Fig. 2:**
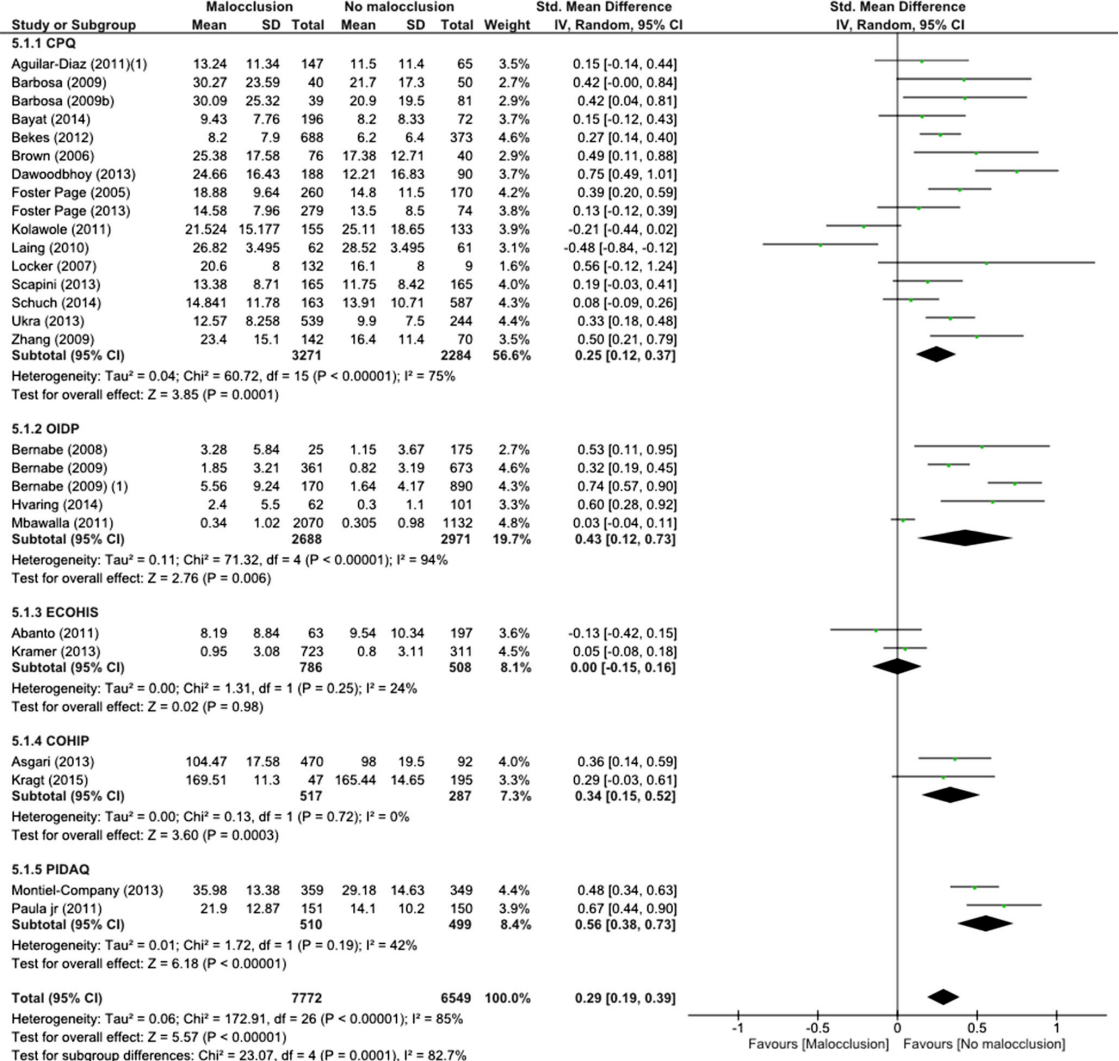
Forest plot and summary measure of the association between malocclusions/orthodontic treatment need and OHRQOL measured with different questionnaires (continuous).

**Fig. 3:**
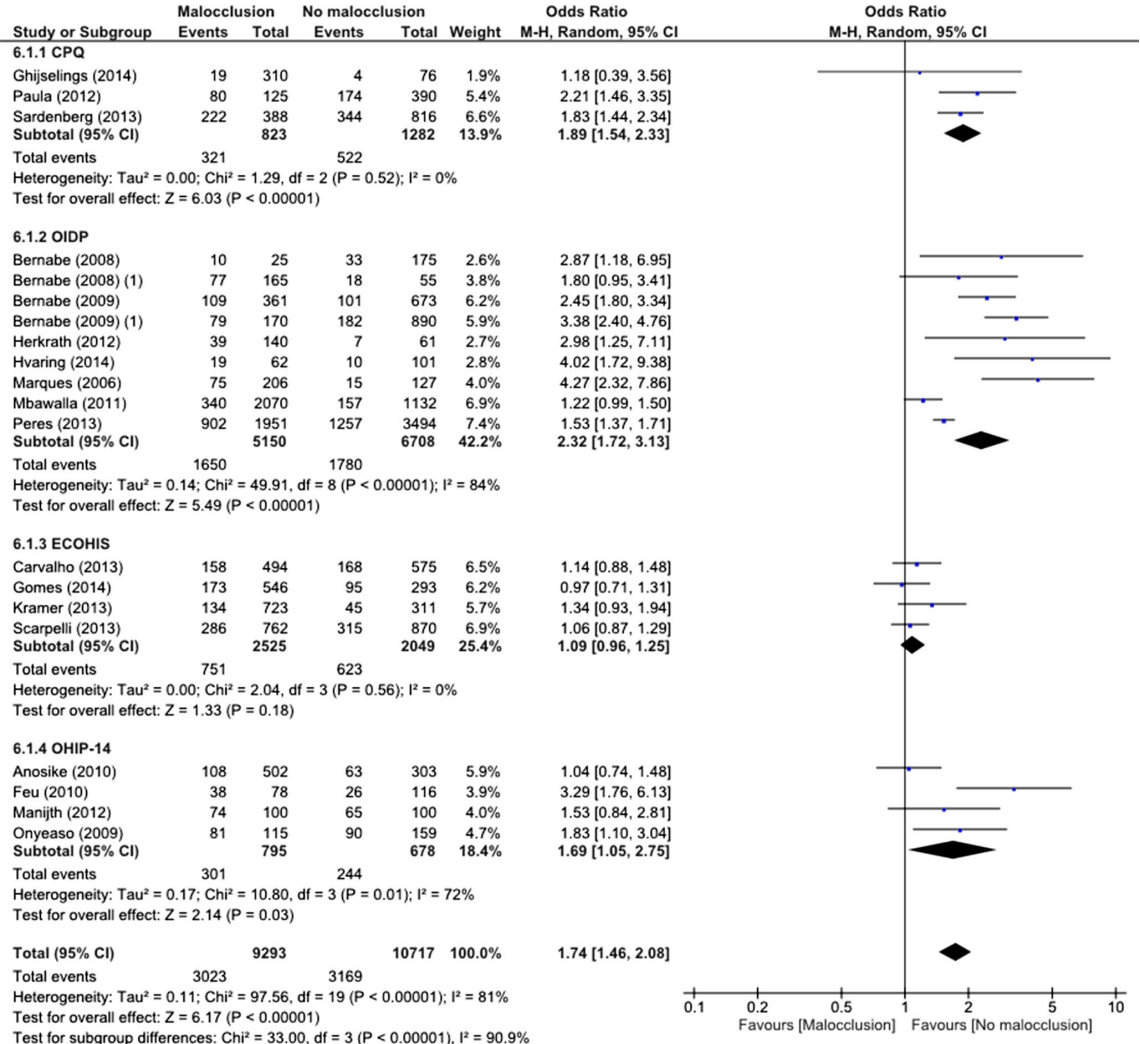
Forest plot and summary measure of the association between malocclusions/orthodontic treatment need and OHRQOL measured with different questionnaires (dichotomous)

In summary, 40 studies, reporting on 41 different samples, were eligible for a quantitative analysis. This resulted in two different meta-analyses, one giving a summary SMD of OHRQOL between children with and children without malocclusions based on 26 studies (Fig. 2) and the other giving a SOR on the impacts of malocclusions on OHRQOL based on 20 studies (Fig. 3). The methodological quality of the individual studies ranged from 3 to 8 points (Supplement S3).

#### Malocclusions and OHRQOL continuously analysed

The summary results show a small but significant SMD in OHRQOL scores between children with malocclusions (*n* = 7772) and without malocclusion (*n* = 6549) (SMD = 0.29, 95 % CI = 0.19–0.39). We observed high heterogeneity (*I* ^2^ = 85 %) among the studies that were combined for the summary measure on malocclusions and OHRQOL scores, which only partly could be explained by the different OHRQOL measures. Although there were significant differences in summary estimates among the different OHRQOL measures (*Χ* ^2^ = 23.07, *p* < 0.001), all indicated a small significant SMD difference in OHRQOL between children with and without malocclusions. Only when OHRQOL was measured with the ECOHIS there was no difference in OHRQOL between children with and without malocclusions (SMD = 0.00, 95 % CI = −0.15–0.16).

#### Malocclusions and OHRQOL dichotomously analysed

The summary result shows that children with malocclusion (*n* = 9293) are 1.74 times more likely to have an impact on OHRQOL than children without malocclusions (*n* = 10,717) (SOR = 1.74, 95 % CI = 1.46–2.08). Again, we observed high heterogeneity (*I* ^2^ = 81 %) among the studies that were combined for the summary measure on the impacts of malocclusions and OHRQOL, which only partly, but more than in the continuous meta-analysis, could be explained by the different OHRQOL measures. The difference in SOR between the different OHRQOL measures was significant (*Χ* ^2^ = 33.00, *p* < 0.001), and again, when OHRQOL was measured with the ECOHIS, no association was found between malocclusions and OHRQOL.

#### Subgroup analyses

We performed several subgroup analyses in both meta-analyses to understand the heterogeneity among the studies assessing the association between malocclusion and OHRQOL. Subgroup analysis based on the method of malocclusion assessment reduced only slightly the heterogeneity in summary estimates, but we found significant differences in summary estimates between the subgroups (continuously, *Χ* ^2^ = 12.92, *df* = 3, *p* = 0.005; dichotomous, *Χ* ^2^ = 18.07, *df* = 4, *p* = 0.001). In the continuous analysis, the association between malocclusion and OHRQOL scores was lost, when malocclusions assessment was based on hypodontia or simply the presence/absence of any malocclusion trait. In contrast, the dichotomous analysis shows that children with malocclusion based on hypodontia are most likely to have any impact on OHRQOL compared to children with malocclusions based on other assessments.

Subgroup analysis based on the age of the participants reduced heterogeneity to a bigger extent, and we found significant differences in summary estimates between the subgroups (continuously, *Χ* ^2^ = 25.98, *df* = 3, *p* < 0.001; dichotomous, *Χ* ^2^ = 27.58, *df* = 3, *p* < 0.001). In the continuous as well as in the dichotomous analysis, we could not see a significant association of malocclusions and OHRQOL in children of age <8 years. Children between 11 and 14 years old were the most likely to have an impact of malocclusions on OHRQOL (SOR = 2.28, 95 % CI = 1.61–3.24), whereas the biggest difference in OHRQOL scores was seen in children older than 14 years old (SMD = 0.59, 95 % CI = 0.40–0.78).

After stratification based on the country of study conduction, we did not found significant differences between subgroups in the continuous meta-analysis, but we did between the subgroups in the dichotomous analysis (continuously, *Χ* ^2^ = 11.50, *df* = 6, *p* = 0.07; dichotomous, *Χ* ^2^ = 13.57, *df* = 4, *p* = 0.009). In general, children with malocclusion were significantly more likely to have lower OHRQOL than children without malocclusions among all countries, except for the studies conducted in Nigeria/Tanzania, where the association based on the continuous analysis goes in the other direction (SMD = −0.06, 95 % CI = −0.30–0.17).

Stratification based on sample recruitment neither reduced heterogeneity nor showed differences between the subgroups.

#### Publication bias

We investigated publication bias visually with funnel plots for both overall meta-analyses. No indication for bias was given. The funnel plots for the continuous and categorical meta-analyses are presented in the supplement (supplement S4).

#### Sensitivity analyses

No or only little differences appeared in the summary estimates, when one of the studies was omitted. None of the changes in the summary estimates were significant (Supplement S5).There were no significant differences in summary estimates between studies of high and low methodological quality (NOS score < 5) in both meta-analyses (Table 2).

**Table 2:** Subgroup analysis of malocclusions/orthodontic treatment need and OHRQOL

Stratum	Subgroup	Continuous (overall hetergoneity 85 %)					Dichotomous (overall hetergoneity 81 %)				
		*N* ^a^	SMD^b^ [95 % CI]	*I* ^2^-statistic^c^	*Χ* ^2^-statistic^d^	*p* value^d^	*N* ^a^	OR [95 % CI]	*I* ^2^-statistic^c^	*Χ* ^2^-statistic^d^	*p* value^d^
Malocclusion	DAI	16	0.37 [0.24, 0.51]	81 %			7	2.24 [1.54, 3.26]	85 %		
	IOTN	4	0.34 [0.24, 0.44]	0 %			4	2.20 [1.55, 3.13]	34 %		
	ICON	–	–	–			1	1.83 [1.10, 3.04]	64 %		
	Hypodontia	2	0.06 [−1.00, 1.12]	95 %			1	4.02 [1.72, 9.38]	–		
	Presence/absence	5	0.09 [−0.03, 0.21]	69 %	12.92	0.005*	7	1.26 [1.06, 1.50]	64 %	18.07	0.001*
Age	<8 years	3	0.04 [−0.08, 0.15]	2 %			4	1.09 [0.96, 1.25]	0.0 %		
	8–10 years	2	0.19 [−0.11, 0.50]	51 %			1	1.83 [1.44, 2.34]	–		
	11–14 years	18	0.28 [0.16, 0.40]	83 %			9	2.28 [1.61, 3.24]	77.0 %		
	>14 years	4	0.59 [0.40, 0.78]	59 %	25.98	<0.001*	6	1.85 [1.30, 2.63]	81.0 %	27.58	<0.001*
Sample	School children	19	0.27 [0.16, 0.38]	86 %			15	1.71 [1.37, 2.14]	84 %		
	Prospective patients	5	0.28 [−0.12, 0.68]	83 %			4	2.30 [1.34, 3.95]	51 %		
	Other	3	0.46 [0.06, 0.86]	82 %	0.77	0.44	1	1.53 [1.37, 1.71]	–	2.63	0.27
Country	Brazil/Mexico	10	0.30 [0.10, 0.51]	87 %			12	1.77 [1.41, 2.23]	85 %		
	New Zealand	3	0.31 [0.18, 0.44]	22 %			–	–	–		
	Canada	1	0.56 [−0.12, 1.24]	–			–	–	–		
	European countries	5	0.25 [0.00, 0.51]	85 %			3	2.58 [1.32, 5.02]	34 %		
	Saudi Arabia/Iran	4	0.44 [0.18, 0.69]	71 %							
	China/Thailand	2	0.36 [0.22, 0.50]	16 %			1	2.45 [1.80, 3.34]	–		
	India	–	–	–			1	1.53 [0.84, 2.81]	–		
	Nigeria/Tanzania	2	−0.06 [−0.30, 0.17]	75 %	11.50	0.07	3	1.25 [0.98, 1.58]	37 %	13.57	0.009*
Methodological quality	Low	8	0.21 [0.06, 0.36]	70 %			4	1.42 [1.02, 1.97]	28 %		
	High	18	0.31 [0.19, 0.44]	85 %	1.05	0.31	16	1.80 [1.47, 2.21]	83 %	1.48	0.22

^a^ *N* number of studies included in subgroups

^b^ *SMD* standardized mean difference

^c^ *I* ^2^-statistic indicated heterogeneity among studies within subgroup

^d^ *X* ^2^-statistic and *p* value indicate differences between subgroups

* indicates significance

## Discussion

In this meta-analysis, we show that malocclusions in children and adolescents between the age of 8 and 18 years are associated with lowered OHRQOL. We clearly see an impact of malocclusions on OHRQOL, albeit this impact seems small. There was high heterogeneity among the studies included in the present meta-analysis, which was partly explained by different factors.

OHRQOL ‘reflects people’s comfort when eating, sleeping and engaging in social interaction, their self-esteem and their satisfaction with respect to their oral health’ [4]. Thus, it encompasses the physical, social and psychological aspects of oral health. Consequently, OHRQOL is suggested to be a multidimensional concept, influenced by individual factors and not stable, but dynamic, over time. This idea is supported by our subgroup analyses, as we show significant differences in the association of malocclusions and OHRQOL among several subgroups.

Firstly, we have shown that the age of the children had a major influence on the association between malocclusions and OHRQOL. Children between the age of 11 and 14, the age when they undergo major life changes, were most likely to have any impact of malocclusions on OHRQOL, but children older than 14 years showed the biggest impact of malocclusions on OHRQOL. In contrast, we did not see any association of malocclusions with OHRQOL in the younger age groups. Correspondingly, we could not see a relationship between malocclusions and the OHRQOL measure designed for and commonly used in toddlers, the ECOHIS [12]. Thus, based on our results it seems that the older the children get, the more their malocclusion affects their OHRQOL and this relation gets at first evident around the children’s age of 8 years old. Longitudinal cohort studies that follow children from the age of 8 years into adulthood would contribute to a better understanding of the dynamics within the relationship of malocclusion with OHRQOL.

Secondly, we also showed differences in the association of malocclusion and OHRQOL between the countries of study conduction, which reflects possible cultural differences. We think that cultural differences may be expressed in both the perception of malocclusions, as well as in the interpretation of OHRQOL. This is in agreement with the World Health Organization Quality of Life group describing the quality of life as an ‘individuals perception of his/her position in life in the context of culture and value systems in which they live […]’ [59]. Also, other authors suggested that the perception of oral health, in this case malocclusion, and its influence on OHRQOL might be influenced by the local health care system, which adds to the explanation of the differences in the association of malocclusion with OHRQOL between countries [60]. Finally, the effect of malocclusions on OHRQOL might depend on how prevalent other oral diseases are and how important dental aesthetics are seen in certain sociocultural structures, which could explain the big difference in the association of malocclusions with OHRQOL between Brazil and African countries. In general, children and their parents may have problems to relate malocclusion to oral health as most orthodontic conditions are asymptomatic [6, 61]. This would explain why we see a clear but relatively small difference in OHRQOL scores between children with malocclusions and children without malocclusions. The size of the overall summary SMD obtained in the present meta-analysis likely reflects changes in one or two questions of OHRQOL measures.

The association of malocclusion and OHRQOL is based on several ideas. Patients with severe or long-term untreated malocclusions might suffer from pain due to temporomandibular disorders or dental trauma [3, 62, 63]. Malocclusion might also cause functional problems, like problems with speaking, mastication and subsequent restricted food choice [3, 64]. Most often, however, researchers write about the impact of malocclusions on the social-emotional domain of OHRQOL. This domain reflects the appearance of the dentition and related bullying, reduced self-esteem related to oral health, and being ashamed of laughing or in interaction with peers [3, 65–67]. We could not investigate these subdomains of OHRQOL individually in this meta-analysis. However, we showed that the associations between malocclusions and OHRQOL varied among the different subgroups of malocclusion assessments. Those assessment methods focus on different aspects of the occlusion, and, therefore, the associations within these subgroups could be translated to a certain domain of OHRQOL. In our meta-analysis, we saw the biggest difference in OHRQOL scores between children with and children without malocclusions, when the latter was assessed with the DAI. The DAI is an orthodontic treatment need index based on socially defined aesthetic standards [68]. This supports, that malocclusions largely impact the social-emotional domain of OHRQOL. In addition, we have seen in our narrative review that some evidence about the association of the IOTN-AC with OHRQOL points in the same direction. Also, our narrative review points to a missing association between the IOTN-DHC or ICON and OHRQOL (Supplement S2). The IOTN-DHC and ICON do measure malocclusion traits that might not be related to the domains of OHRQOL, like crossbites or impacted teeth in an early stage.

This is the first meta-analysis on the association of malocclusions or orthodontic treatment need and OHRQOL in children and adolescents. An important factor in meta-analysis is the quality of the included studies. We did not exclude studies based on their methodological quality, because our main aim was to give a complete comprehensive overview of the topic. All studies in this meta-analysis were cross-sectional, which is considered to be the study design of the lowest quality because of its susceptibility to reverse causation. However, reverse causation is not a matter of concern to the association of malocclusion with OHRQOL. In addition, we evaluated the methodological quality of the individual studies and we did not find significant differences between studies of high and low quality. Generally, we extracted the descriptive data from the selected articles. Therefore, our data are all crudely analysed, without adjustments for confounders like gender, social economic status (SES) or other oral diseases. However, in this way, we were able to include a maximum of studies. We also did not adjust the results for whether the OHRQOL instruments address the questions directly to the children (OIDP, CPQ, OHIP) or make use of parent forms (ECOHIS, COHIP). Accordance between parental and child reports on OHRQOL is widely described in the literature. Especially in orthodontics, parents are seen as valid proxies for the assessment of their children’s OHRQOL [69–72]. If discrepancies between parents and children assessments exist, children tend to report their own OHRQOL lower than their caregivers do, which means that the associations between children’s malocclusion and OHRQOL assessed by parents are rather underestimated than overestimated. Though, we conducted the meta-analysis on 40 studies including 28,496 children and, therefore, we think that the benefits of the quantitative analysis outweigh the limitations of this meta-analysis.

Several systematic reviews about malocclusions and OHRQOL have been conducted; however, to our knowledge, only two have restricted their studies to children and adolescents [2, 3, 6, 7, 73]. Barbosa and Gaviaõ wrote about contradictory results among six studies on the association of malocclusions and OHRQOL [6]. Dimberg et al have recently published a systematic review on the association of malocclusions and OHRQOL and tried to limit variability by restricting their review to high-quality studies (*n* = 6) [73]. In both reviews, the researchers suggest that the effect of malocclusions is mainly on the social-emotional wellbeing domain, but they can only speculate on other sources of inconsistency [6, 73]. The strength of our study is that we are able to explain some sources of this variability in the association between malocclusion and OHRQOL. Another strength of our study is that we analysed both dichotomous and continuous data on the association of malocclusions and OHRQOL. In this way, we maximized the amount of included studies. In addition, we could not only write about whether there is an impact of malocclusions on OHRQOL, but we make conclusion about the size of the impact. Finally, we have also shown that the results of our summary measures are robust and have not been affected by publication bias.

Unfortunately, in this systematic review, we were not able to focus on more personal factors influencing the association of malocclusions with OHRQOL, like SES, gender or self-esteem. Only one study, included in the narrative review, noticed that the association between malocclusion and OHRQOL is attenuated in children with low self-esteem [74]. This might be one reason why we could not explain all heterogeneity among the studies. However, we noticed increasing research interest in the modifying role of personal factors in the association of malocclusions with OHRQOL, and based on this meta-analysis, we highly recommend to continue this research strand.

## Conclusion

The association of malocclusion and OHRQOL has mainly been assessed in cross-sectional studies. From these studies, it can be concluded that children perceive a small impact of malocclusions on OHRQOL. The effect of malocclusions on OHRQOL is modified by the age of the children and their cultural environment. Further research should investigate whether remaining heterogeneity in the association of malocclusions with OHRQOL can be explained by other individual factors of the children.

## Electronic supplementary material

Below is the link to the electronic supplementary material.

ESM 1 (PDF 238 kb)
