# Supplementary material for: The impact of malocclusions on oral health-related quality of life in children—a systematic review and meta-analysis
Source: Clin Oral Investig. 2015 Dec 4;20(8):1881–94. doi: 10.1007/s00784-015-1681-3 (PMC5069349; doi:10.1007/s00784-015-1681-3)
Supplement: Supplementary file 1 — (PDF 238 kb) [file 784_2015_1681_MOESM1_ESM.pdf]

Supplemental file

Title:

THE IMPACT OF MALOCCLUSIONS ON ORAL HEALTH RELATED QUALITY OF LIFE IN CHILDREN - A SYSTEMATIC LITERATURE REVIEW AND META-ANALYSIS

Authors:

Lea Kragt, MSc; Department of Oral & Maxillofacial Surgery, Special Dental Care and Orthodontics, Erasmus Medical Center, Rotterdam, The Netherlands; Department of The Generation R Study Group, Erasmus University Medical Centre, Rotterdam, The Netherlands; l.kragt@erasmusmc.nl

Brunilda Dhamo, DDs, MSc; Department of Oral & Maxillofacial Surgery, Special Dental Care and Orthodontics, Erasmus Medical Center, Rotterdam, The Netherlands; Department of The Generation R Study Group, Erasmus University Medical Centre, Rotterdam, The Netherlands; bruna18mars@yahoo.it

Eppo B. Wolvius, DDS, MD, PhD, Prof; Department of Oral & Maxillofacial Surgery, Special Dental Care and Orthodontics, Erasmus Medical Center, Rotterdam, The Netherlands; Department of The Generation R Study Group, Erasmus University Medical Centre, Rotterdam, The Netherlands; e.wolvius@erasmusmc.nl

Edwin M.Ongkosuwito, DDS, PhD; Department of Oral & Maxillofacial Surgery, Special Dental Care and Orthodontics, Erasmus Medical Center, Rotterdam, The Netherlands; Department of The Generation R Study Group, Erasmus University Medical Centre, Rotterdam, The Netherlands; e.ongkosuwito@erasmusmc.nl

Journal name: Clinical Oral Investigations

1. Supplement S1

Search Strategy

Embase

('quality of life'/exp OR 'self concept'/exp OR 'psychological well being'/de OR ((qualit* NEAR/3 life) OR QoL OR HRQL OR HRQoL OR OHRQL OR OHRQoL OR ((health OR malocclus* OR occlus* OR overbite OR underbite OR emotion* OR aesthet* OR esthet* OR appear* OR attractive* OR 'facial profile' OR psycholog*) NEAR/3 (impact OR percept* OR perciev* OR aware* OR satisf* OR dissatisf*)) OR (self NEXT/1 (concept* OR esteem* OR percept* OR perceiv*)) OR 'personal appearance' OR 'well being' OR wellbeing OR (social NEAR/3 function*) OR ((desire* OR demand* OR need OR needs OR motivat*) NEAR/6 (treatment* OR intervention*))):ab,ti) AND (orthodontics/de OR malocclusion/de OR (orthodont* OR (occlus* NEAR/3 adjust*) OR malocclusion* OR (palat* NEAR/3 expan*) OR (bite NEAR/3 correction*) OR (serial NEAR/3 extraction*) OR overbite OR underbite OR (occlus* NEAR/3 disorder*)):ab,ti)

Medline OvidSP

("quality of life"/ OR exp "self concept"/ OR "Personal Satisfaction"/ OR ((qualit* ADJ3 life) OR QoL OR HRQL OR HRQoL OR OHRQL OR OHRQoL OR ((health OR malocclus* OR occlus* OR overbite OR underbite OR emotion* OR aesthet* OR esthet* OR appear* OR attractive* OR "facial profile" OR psycholog*) ADJ3 (impact OR percept* OR perciev* OR aware* OR satisf* OR dissatisf*)) OR (self ADJ (concept* OR esteem* OR percept* OR perceiv*)) OR "personal appearance" OR "well being" OR wellbeing OR (social ADJ3 function*) OR ((desire* OR demand* OR need OR needs OR motivat*) ADJ6 (treatment* OR intervention*))).ab,ti.) AND (orthodontics/ OR exp "Orthodontics, Corrective"/ OR exp malocclusion/ OR

(orthodont* OR (occlus* ADJ3 adjust*) OR malocclusion* OR (palat* ADJ3 expan*) OR (bite ADJ3 correction*) OR (serial ADJ3 extraction*) OR overbite OR underbite OR (occlus* ADJ3 disorder*)).ab,ti.)

Cochrane central
(((qualit* NEAR/3 life) OR QoL OR HRQL OR HRQoL OR OHRQL OR OHRQoL OR ((health OR malocclus* OR occlus* OR overbite OR underbite OR emotion* OR aesthet* OR esthet* OR appear* OR attractive* OR 'facial profile' OR psycholog*) NEAR/3 (impact OR percept* OR perciev* OR aware* OR satisf* OR dissatisf*)) OR (self NEXT/1 (concept* OR esteem* OR percept* OR perceiv*)) OR 'personal appearance' OR 'well being' OR wellbeing OR (social NEAR/3 function*) OR ((desire* OR demand* OR need OR needs OR motivat*) NEAR/6 (treatment* OR intervention*))):ab,ti) AND ((orthodont* OR (occlus* NEAR/3 adjust*) OR malocclusion* OR (palat* NEAR/3 expan*) OR (bite NEAR/3 correction*) OR (serial NEAR/3 extraction*) OR overbite OR underbite OR (occlus* NEAR/3 disorder*)):ab,ti)

Web-of-science
TS=((((qualit* NEAR/3 life) OR QoL OR HRQL OR HRQoL OR OHRQL OR OHRQoL OR ((health OR malocclus* OR occlus* OR overbite OR underbite OR emotion* OR aesthet* OR esthet* OR appear* OR attractive* OR "facial profile" OR psycholog*) NEAR/3 (impact OR percept* OR perciev* OR aware* OR satisf* OR dissatisf*)) OR (self NEAR/1 (concept* OR esteem* OR percept* OR perceiv*)) OR "personal appearance" OR "well being" OR wellbeing OR (social NEAR/3 function*) OR ((desire* OR demand* OR need OR needs OR motivat*) NEAR/6 (treatment* OR intervention*)))) AND ((orthodont* OR (occlus* NEAR/3 adjust*) OR malocclusion* OR (palat* NEAR/3 expan*) OR (bite NEAR/3 correction*) OR (serial NEAR/3 extraction*) OR overbite OR underbite OR (occlus* NEAR/3 disorder*)))) AND DT=(Article)

Scopus
TITLE-ABS-KEY((((qualit* W/3 life) OR QoL OR HRQL OR HRQoL OR OHRQL OR OHRQoL OR ((health OR malocclus* OR occlus* OR overbite OR underbite OR emotion* OR aesthet* OR esthet* OR appear* OR attractive* OR "facial profile" OR psycholog*) W/3 (impact OR percept* OR perciev* OR aware* OR satisf* OR dissatisf*)) OR (self PRE/1 (concept* OR esteem* OR percept* OR perceiv*)) OR "personal appearance" OR "well being" OR wellbeing OR (social W/3 function*) OR ((desire* OR demand* OR need OR needs OR motivat*) W/6 (treatment* OR intervention*)))) AND ((orthodont* OR (occlus* W/3 adjust*) OR malocclusion* OR (palat* W/3 expan*) OR (bite W/3 correction*) OR (serial W/3 extraction*) OR overbite OR underbite OR (occlus* W/3 disorder*)))) AND DOCTYPE(ar)

PsycINFO OvidSP
("quality of life"/ OR "Life Satisfaction"/ OR "Well Being"/ OR exp "self concept"/ OR "Self Perception"/ OR exp "Satisfaction"/ OR Dissatisfaction/ OR ((qualit* ADJ3 life) OR QoL OR HRQL OR HRQoL OR OHRQL OR OHRQoL OR ((health OR malocclus* OR occlus* OR overbite OR underbite OR emotion* OR aesthet* OR esthet* OR appear* OR attractive* OR "facial profile" OR psycholog*) ADJ3 (impact OR percept* OR perciev* OR aware* OR satisf* OR dissatisf*)) OR (self ADJ (concept* OR esteem* OR percept* OR perceiv*)) OR "personal appearance" OR "well being" OR wellbeing OR (social ADJ3 function*) OR ((desire* OR demand* OR need OR needs OR motivat*) ADJ6 (treatment* OR intervention*))).ab,ti.) AND ((orthodont* OR (occlus* ADJ3 adjust*) OR malocclusion* OR (palat* ADJ3 expan*) OR (bite ADJ3 correction*) OR (serial ADJ3 extraction*) OR overbite OR underbite OR (occlus* ADJ3 disorder*)).ab,ti.) NOT book.pt.

PubMed publisher
((quality of life*[tiab] OR QoL[tiab] OR HRQL[tiab] OR HRQoL[tiab] OR OHRQL[tiab] OR OHRQoL[tiab] OR ((malocclus*[tiab] OR occlus*[tiab] OR overbite[tiab] OR underbite[tiab] OR emotion*[tiab] OR aesthet*[tiab] OR esthet*[tiab] OR appear*[tiab] OR attractive*[tiab] OR facial profile OR psycholog*[tiab]) AND (impact[tiab] OR percept*[tiab] OR perciev*[tiab] OR aware*[tiab] OR satisf*[tiab] OR dissatisf*[tiab])) OR self concept*[tiab] OR self esteem*[tiab] OR self percept*[tiab] OR self perceiv*[tiab] OR personal appearan*[tiab] OR well being*[tiab] OR wellbeing[tiab] OR social function*[tiab] OR ((desire*[tiab] OR demand*[tiab] OR need[tiab] OR needs[tiab] OR motivat*[tiab]) AND (treatment*[tiab] OR

intervention*[tiab])))) AND ((orthodont*[tiab] OR (occlus*[tiab] AND adjust*[tiab]) OR malocclusion*[tiab] OR (palat*[tiab] AND expan*[tiab]) OR (bite AND correction*[tiab]) OR serial extraction*[tiab]) OR overbite[tiab] OR underbite[tiab] OR (occlus*[tiab] AND disorder*[tiab]))) AND publisher[sb]

Cinahl
(MH "quality of life+" OR MH "self concept+" OR MH "Personal Satisfaction+" OR ((qualit* N3 life) OR QoL OR HRQL OR HRQoL OR OHRQL OR OHRQoL OR ((health OR malocclus* OR occlus* OR overbite OR underbite OR emotion* OR aesthet* OR esthet* OR appear* OR attractive* OR "facial profile" OR psycholog*) N3 (impact OR percept* OR perciev* OR aware* OR satisf* OR dissatisf*)) OR (self N1 (concept* OR esteem* OR percept* OR perceiv*)) OR "personal appearance" OR "well being" OR wellbeing OR (social N3 function*) OR ((desire* OR demand* OR need OR needs OR motivat*) N6 (treatment* OR intervention*)))) AND (MH orthodontics+ OR MH "Orthodontics, Corrective+" OR MH malocclusion+ OR (orthodont* OR (occlus* N3 Nust*) OR malocclusion* OR (palat* N3 expan*) OR (bite N3 correction*) OR (serial N3 extraction*) OR overbite OR underbite OR (occlus* N3 disorder*)))

Google Scholar
"quality of life"|"self concept"|psychological|"well being"|wellbeing|impact|awareness|satisfaction|dissatisfaction|appearance orthodontics|"occlusive|occlusion adjustment|disorder"|malocclusion|"bite correction"|"serial extraction"|overbite|underbite

2. **Supplement S2**

Table S1 Study characteristics of studies included in the narrative review but excluded from the meta-analysis (N=17)

	**Author**	**Year**	**Country**	**Study type**	**Participants**	**Age (Mean)**	**N**	**OHRQOL**	**Malocclusion**	**Ref**
1	Abanto, J.et al.	2014	Brasil	cross-sectional	Pre-school children	2.53	879	ECOHIS	Presence/Absence	[1]
2	Agou, S. et al	2008	Canada	cross-sectional	Children seeking orthodontic treatment	12.6	191	CPQ11-14	DAI	[2]
3	Aguilar-Diaz, F.C. et al.	2011	Mexico	cross-sectional	Schoolchildren	8.8	315	CPQ8-10	DAI	[3]
4	Ahn,Y.S. et al.	2012	Korea	cross-sectional	Korean Oral Health Survey 2010	11.8	2236	COHIP	IOTN	[4]
5	Bianco, A. et al.	2010	Italy	cross-sectional	Students from secondary schools, Catanzaro	-	530	child OIDP	IOTN-AC	[5]
6	Castro, R.D.A.L. et al.	2011	Brasil	cross-sectional	Students from public schools, Rio de Janeiro	12.0	571	child OIDP	Presence/Absence	[6]
7	De Baets, E. et al.	2012	Belgium	cross-sectional	Healthy child registered for a first consultation at the Orthodontic Department	13.22	223	CPQ11-14	IOTN-DHC/ -AC	[7]
8	de Oliveira C.M. et al.	2008	United Kingdom	cross-sectional	Referred to orthodontic clinical	12.21	187	OIDP	IOTN-DHC	[8]
9	de Paula J.S. et al.	2013	Brasil	cross-sectional	School children	-	286	CPQ11-14	DAI	[9]
10	Do, et al.	2008	Australia	cross-sectional	nested on cohort	-	842	CPQ8-10/CPQ11-14	DAI	[10]
11	Heravi, et al.	2011	Iran	cross-sectional	High school students	-	120	CPQ	ICON	[11]
12	Johal, A. et al.	2007	United Kingdom	cross-sectional	-	-	90	CPQ11-14	overjet/ spacing	[12]
13	Kotecha, et al	2013	United Kingdom	cross-sectional	clinical sample + comparison	12.5	116	CPQ11-14	Hypodontia	[13]
14	Marshman, et al.	2005	United Kingdom	cross-sectional	Consecutive sample	12.4	89	CPQ11-14	IOTN	[14]
15	Motamedi, M.R.K. et al.	2014	Iran	cross-sectional	Schoolchildren	13.0	336	CPQ11-14	IOTN	[15]
16	Sousa, R. V. et al.	2014	Brasil	cross-sectional	Preschool children	3.9	732	ECOHIS	Overbite, -jet, crossbite	[16]
17	Thelen, D.S. et al.	2011	Norway	cross-sectional	Adolescents in schools	17.2	493	OIDP	IOTN-AC	[17]

Narrative review of studies not included in meta-analysis

Of all 57 studies, 17 studies could not be included in the meta-analysis, because the mean OHRQOL score was not reported [1, 2, 7-9, 12-15], the number of cases per subgroup were missing [3-6, 10, 11, 16], or orthodontic treatment need was only assessed with the IOTN-AC [5, 7, 15, 17].

Malocclusion assessment and OHRQOL measures of studies included in meta-analysis

The most commonly (n = 10) used OHRQOL questionnaires were the two Child Perception Questionnaires (CPQ), i.e. for the age group 8-10 years [3, 10] and the age group 11-14 years [2, 7, 9-15]. Also the Oral Impacts of Daily Performances (OIDP) was often used (n = 4) [5, 6, 8, 17]. Two studies used the Early Childhood Oral Health Scale (ECOHIS) [1, 16].One study used the Child Oral health impact profile (COHIP) [4].

Studies used several methods to assess malocclusions or orthodontic treatment need in their study population. Most of the time (n= 5) the IOTN-DHC was used [4, 7, 8, 14, 15]. The DAI was used in four studies [2, 3, 14, 15]. The ICON was used in one study [11]. Four studies only assessed presence of any malocclusion trait or anterior malocclusion trait . One study assessed hypodontia [13].

Nine studies used the Aesthetic Component of the Index of Orthodontic treatment need (IOTN-AC), either exclusive or in combination with the IOTN-DHC [5, 7, 15, 17-22].

Narrative review

All studies that investigated the association between malocclusions measured with the DAI and OHRQOL measured by the CPQ found significant lower OHRQOL in children with malocclusions [2, 3, 9, 10]. However, these association were mostly weak and one study showed that this association between malocclusion based on DAI scores and CPQ scores was lost in children with low self-esteem [2]. All studies that investigated the association between malocclusions measured with the IOTN-DHC and OHRQOL measured by the CPQ found no relationship between OHRQOL and orthodontic treatment need (IOTN-DHC >3) [7, 8, 14, 15]. Only one study investigated additionally the correlation between IOTN scores and CPQ scores, which was significant but weak [7]. One study investigated the relationship of malocclusions measured with the ICON and showed only non-significant higher OHRQOL scores (CPQ) in the severe and moderate group compared to the acceptable group [11]. Johal et al found highly statistical significant differences between groups with either an

increased overjet or spaced dentition compared to a control group [12]. Finally, one study investigated the relationship between tooth agenesis with OHRQOL measured by the CPQ. Tooth agenesis was associated with lower OHRQOL, however there was no correlation with the number of missing teeth [13].

Two studies that were not included in the meta-analysis used the ECOHIS to assess OHRQOL in children up to 5 years old [1, 16]. Both studies investigated the relationship between the presence of malocclusion and ECOHIS scores as well as kind of malocclusion with ECOHIS scores, but no significant association between any malocclusion trait and children's OHRQOL was found [16, 23].

One study used the COHIP to assess OHRQOL in children. Ahn et al. related the IOTN-DHC scores with OHRQOL measured by the COHIP and found significant but little worse OHRQOL in children with orthodontic treatment need (IOTN-DHC > 3) [4]. Castro et al used the OIDP to assess OHRQOL in children and found an significant association between inadequate position of the teeth with lower OHRQOL (OIDP > 0) [6].

In total, 4 articles of the studies that were not included in the meta-analyses investigated the relationship between the IOTN-AC and OHRQOL in children [5, 7, 15, 17]. Of these, three article did not find a relationship between IOTN-AC scores and OHRQOL measured with the CPQ [15] or the OIDP [5, 17]. One article found a significant but weak association between the IOTN-AC and OHRQOL measured with the CPQ [7]. In contrast, the studies that were included into the meta-analyses and assessed the IOTN-AC found significant relations between the IOTN-AC scores and OHRQOL [19, 22], except Kragt et al. who did only find a borderline significant relation [20].

3. Supplement S3

Table 2S Quality assessment of studies included in the meta-analysisbased on the Newcastle-Ottawa Scale adapted for cross-sectional studies

		**Selection:**				**Comparability**	**Outcome**		**Score**
Artikel	***Ref***	***Sample representativeness***	***SS***	***Non respondents***	***Exposure assessment***	***Control for differences***	***Outcome assessment***	***Statistics***	
Abanto, J.et al.	[23]	0	0	1	1	1	1	1	5/10
Aguilar-Diaz, F.C. et al.	[24]	0	0	1	2	1	1	1	6/10
Anosike, A.N. et al.	[25]	1	0	1	2	0	1	1	6/10
Asgari, I. et al.	[26]	1	1	1	2	0	1	1	7/10
Barbosa, T.S. et al.	[27]	1	0	0	2	0	1	1	5/10
Bhayat, A. et al.	[28]	1	0	1	1	0	1	1	5/10
Bekes, K. et al.	[29]	1	0	0	1	0	1	0	3/10
Bernabe, E. et al.	[30]	1	1	1	2	1	1	1	8/10
Bernabe, E. et al. (1)	[31]	1	1	0	1	1	1	1	6/10
Bernabe, E. et al. (1)	[32]	1	1	0	2	1	1	1	7/10
Bernabe, E. et al.	[33]	0	1	1	2	1	1	1	7/10
Brown A. et al.	[34]	1	0	1	1	0	1	1	5/10
Carvalho, A.C. et al.	[35]	1	1	1	1	1	1	1	7/10
Dawoodbhoy, I. et al.	[36]	1	1	0	2	0	1	1	6/10
Feu D. et al.	[18]	1	1	1	2	1	1	1	8/10
Foster Page, L.A. et al.	[37]	1	1	1	2	0	1	1	7/10
Foster Page, L.A. et al.	[38]	1	0	0	2	1	1	1	6/10
Ghijselings, I. et al.	[19]	1	1	0	2	0	1	0	5/10
Gomes, M.C. et al.	[39]	1	1	1	1	1	1	1	7/10
Herkrath, F.J. et al.	[40]	1	1	0	1	0	1	1	5/10
Hvaring, C.L. et al.	[41]	0	1	1	2	1	1	1	7/10
Kolawole, et al.	[42]	1	1	1	2	0	1	1	7/10
Kragt, et al.	[20]	1	0	1	2	0	1	1	7/10
Kramer, P.F. et al.	[43]	1	0	1	1	1	1	1	7/10
Laing, E. et al.	[44]	0	1	0	2	1	1	1	6/10
Locker, D. et al.	[45]	0	0	0	1	0	1	1	3/10
Manijth, C.M. et al.	[46]	0	0	0	2	1	1	0	4/10
Marques, L.S. et al.	[47]	1	1	1	2	0	1	1	7/10
Mbawalla, H.S. et al.	[48]	0	1	0	1	1	1	1	5/10
Montiel-Company, J.M. et al.	[22]	1	0	0	2	1	1	1	6/10
Onyeaso, et al.	[49]	1	1	0	2	1	1	1	7/10
Paula, J.S. et al.	[50]	1	1	0	2	1	1	1	7/10
Paula Jr, et al.	[51]	1	0	0	2	1	1	1	6/10
Peres, K.G. et al.	[52]	1	0	1	2	1	1	1	7/10
Sardenberg F. et al.	[53]	1	1	0	2	1	1	1	7/10
Scapini, A. et al.	[54]	1	1	1	2	1	1	1	8/10
Scarpelli, A.C. et al.	[55]	1	1	0	1	1	1	1	6/10
Schuch, H.S. et al.	[56]	1	1	0	2	0	1	1	6/10
Ukra, A. et al.	[57]	1	0	0	2	1	1	1	6/10
Zhang, M. et al.	[21]	1	0	1	2	1	0	1	6/10

4. Supplement S4

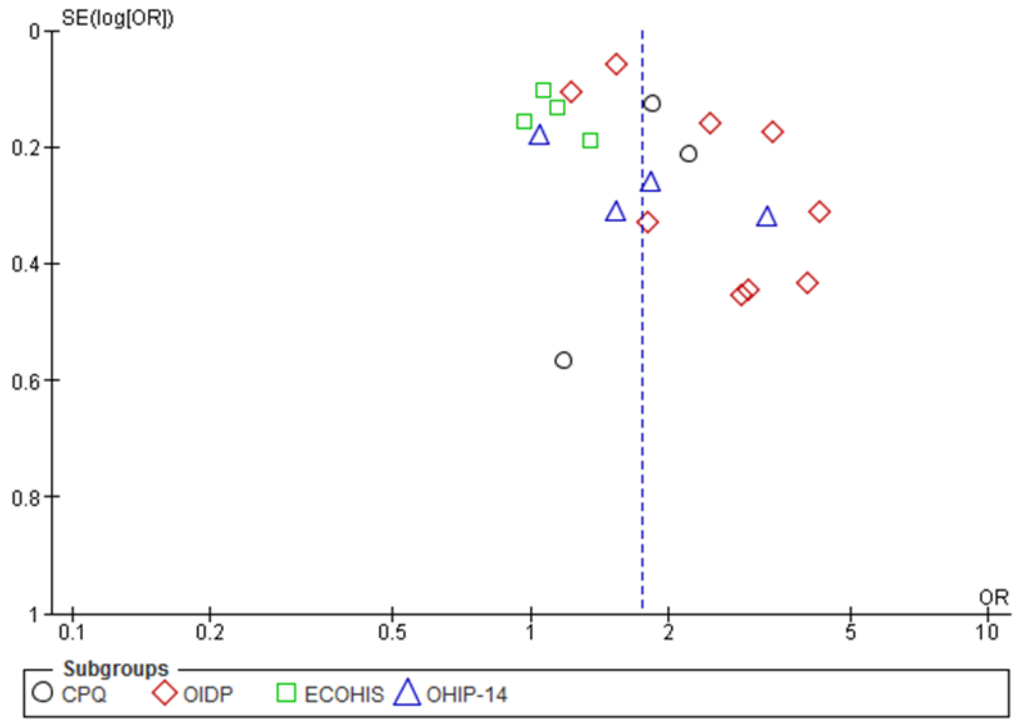

Figure S1 Funnel plot fort he evaluation of publication bias for studies evaluating the association of malocclusion and OHRQOL categorically

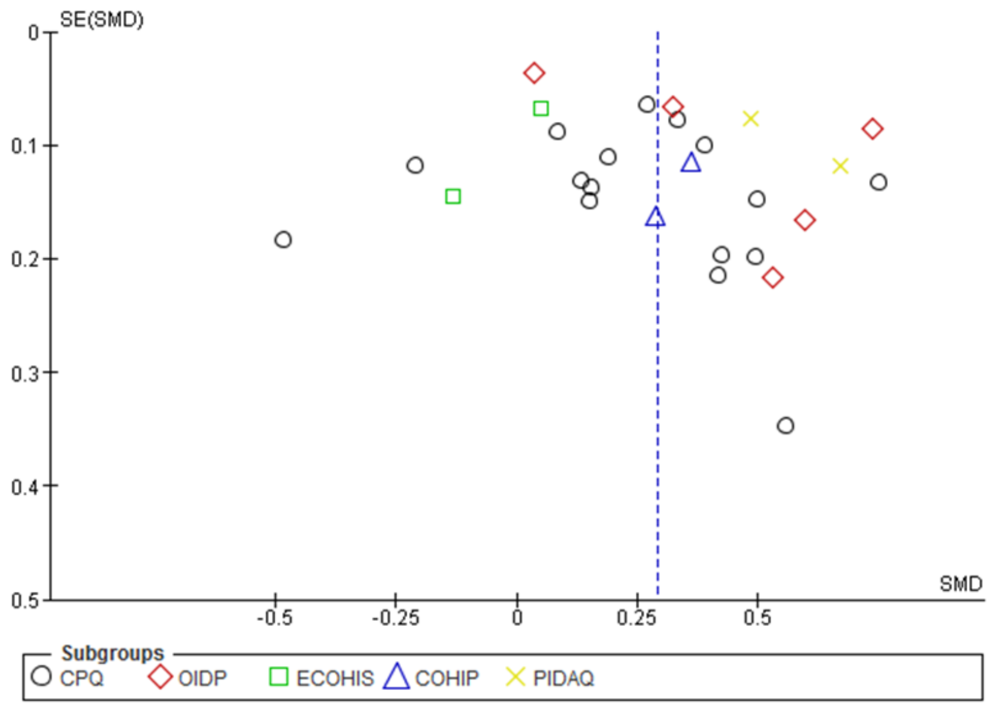

Figure S2 Funnel plot fort he evaluation of publication bias for studies evaluating the association of malocclusion and OHRQOL dichotomously

5. Supplement S5

Table S3 Sensitivity analysis for the different meta-analyses about OHRQOL and malocclusions

	OHRQOL	Study Omitted	Ref	SMD / OR [95% CI]	P-value for difference
continuous	CPQ[1]	Aguilar-Diaz (2011) (1)	[24]	0.30 [0.19, 0.40]	0.892
		Barbosa (2009)	[27]	0.29 [0.18, 0.39]	1.000
		Barbosa (2009b)	[27]	0.29 [0.18, 0.39]	1.000
		Bhayat (2014)	[28]	0.30 [0.19, 0.40]	0.892
		Bekes (2012)	[29]	0.29 [0.18, 0.40]	1.000
		Brown (2006)	[34]	0.28 [0.18, 0.39]	0.892
		Dawoodbhoy (2013)	[36]	0.27 [0.17, 0.37]	0.782
		Foster Page (2005)	[37]	0.29 [0.18, 0.39]	1.000
		Foster Page (2013)	[38]	0.30 [0.19, 0.40]	0.892
		Kolawole (2011)	[42]	0.31 [0.21, 0.41]	0.782
		Laing (2010)	[44]	0.31 [0.21, 0.41]	0.782
		Locker (2007)	[45]	0.29 [0.18, 0.39]	1.000
		Scapini (2013)	[54]	0.30 [0.19, 0.40]	0.892
		Schuch (2014)	[56]	0.30 [0.19, 0.41]	0.895
		Ukra (2013)	[57]	0.29 [0.18, 0.40]	1.000
		Zhang (2009)	[21]	0.28 [0.18, 0.39]	0.892
	OIDP[2]	Bernabe (2008)	[30]	0.28 [0.18, 0.39]	0.892
		Bernabe (2009)	[33]	0.29 [0.18, 0.40]	1.000
		Bernabe (2009) (1)	[32]	0.27 [0.17, 0.37]	0.782
		Hvaring (2014)	[41]	0.28 [0.18, 0.38]	0.890
		Mbawalla (2011)	[48]	0.30 [0.20, 0.41]	0.892
	ECOHIS[3]	Abanto (2011)	[23]	0.31 [0.20, 0.41]	0.787
		Kramer (2013)	[43]	0.30 [0.20, 0.41]	0.892
	COHIP[4]	Asgari (2013)	[26]	0.29 [0.18, 0.39]	1.000
		Kragt (2015)	[20]	0.29 [0.19, 0.40]	1.000
	PIDAQ[5]	Montiel-Company (2009)	[22]	0.28 [0.18, 0.39]	0.892
		Paula jr (2011)	[51]	0.27 [0.17, 0.38]	0.787
dichotomous	CPQ	Ghijselings (2014)	[19]	1.76 [1.47, 2.10]	0.929
		Paula (2012)	[50]	1.72 [1.43, 2.06]	0.929
		Sardenberg (2013)	[53]	1.74 [1.44, 2.10]	1.000
	OIDP	Bernabe (2008)	[30]	1.72 [1.44, 2.06]	0.929
		Bernabe (2008) (1)	[31]	1.74 [1.45, 2.09]	1.000
		Bernabe (2009)	[33]	1.70 [1.42, 2.03]	0.857
		Bernabe (2009)	[33]	1.65 [1.40, 1.95]	0.859
		Herkrath (2012)	[40]	1.72 [1.44, 2.05]	0.856
		Hvaring (2014)	[41]	1.70 [1.42, 2.03]	0.857
		Marques (2006)	[47]	1.67 [1.41, 1.98]	0.852
		Mbwalla (2011)	[48]	1.80 [1.49, 2.17]	0.798
		Peres (2013)	[52]	1.79 [1.45, 2.21]	0.810
	ECOHIS	Carvalho (2013)	[35]	1.80 [1.49, 2.16]	0.797
		Gomes (2014)	[39]	1.81 [1.51, 2.16]	0.760
		Kramer (2013)	[43]	1.77 [1.47, 2.14]	0.897
		Scarpelli (2013)	[55]	1.81 [1.51, 2.17]	0.762
	OHIP-14	Anosike (2010)	[25]	1.87 [1.56, 2.26]	0.614
		Feu (2010)	[18]	1.76 [1.46, 2.11]	0.930
		Manjith (2012)	[46]	1.82 [1.51, 2.20]	0.735
		Onyeaso (2009)	[49]	1.81 [1.50, 2.18]	0.766

References

1. Abanto J, Tello G, Bonini GC, Oliveira LB, Murakami C, Bönecker M (2014) Impact of traumatic dental injuries and malocclusions on quality of life of preschool children: a population-based study. Int J Paediatr Dent **25**: 18-28.
2. Agou S, Locker D, Streiner DL, Tompson B (2008) Impact of self-esteem on the oral-health-related quality of life of children with malocclusion. Am J Orthod Dentofacial Orthop **134**: 484-489.
3. Aguilar-Diaz FC, Irigoyen-Camacho M (2011) Validation of the CPQ 8-10ESP in Mexican school children in urban areas. Med Oral Patol Oral Cir Bucal **16**: 430-435.
4. Ahn YS, Kim HY, Hong SM, Patton LL, Kim JH, Noh HJ (2012)Validation of a Korean version of the Child Oral Health Impact Profile (COHIP) among 8- to 15-year-old school children. Int J Paediatr Dent **22**: 292-301.
5. Bianco A, Fortunato L, Nobile CGA, Pavia M (2010) Prevalence and determinants of oral impacts on daily performance: results from a survey among school children in Italy. Eur J Public Health **20**: 595-600.
6. Castro RAL, Portela MC, Leão AT, de Vasconcellos MTL (2011) Oral health-related quality of life of 11- and 12-year-old public school children in Rio de Janeiro. Community Dent Oral Epidemiol **39**: 336-344.
7. De Baets E, Lambrechts H, Lemiere J, Diya L, Willems G (2012) Impact of self-esteem on the relationship between orthodontic treatment need and oral health-related quality of life in 11- to 16-year-old children. Eur J Orthod **34**: 731-737.
8. De Oliveira CM, Sheiam A, Tsakos G, O'Brien KD (2008) Oral health-related quality of life and the IOTN index as predictors of children's perceived needs and acceptance for orthodontic treatment. Brit Dent J **204**: 1-5.
9. de Paula JS, Leite ICG, de Almeida AB, Ambrosano GMB, Mialhe FL (2013) The impact of socioenvironmental characteristics on domains of oral health-related quality of life in Brazilian schoolchildren. Bmc Oral Health **13**: 10.
10. Do LG, Spencer AJ (2008) Evaluation of oral health-related quality of life questionnaires in a general child population. Community Dent Health **25**: 205-210.
11. Heravi F, Farzanegan F, Tabatabaee M, Sadeghi M (2011) Do malocclusions affect the oral health-related quality of life? Oral Health Prev Dent **9**: 229-233.
12. Johal A, Cheung MY, Marcene W (2007) The impact of two different malocclusion traits on quality of life. Br Dent J **202**: E2.
13. Kotecha S, Turner PJ, Dietrich T, Dhopatkar A (2013) The impact of tooth agenesis on oral health-related quality of life in children. J Orthod **40**: 122-129.
14. Marshman Z, Rodd H, Stern M, Mitchell C, Locker D, Jokovic A, Robinson PG (2005) An evaluation of the Child Perceptions Questionnaire in the UK. Community Dent Health **22**: 151-155.
15. Motamedi MRK, Behzadi A, Khodadad N, Zadeh AK, Nilchian F (2014) Oral health and quality of life in children: A cross-sectional study. Dental Hypotheses **5**: 53-58.
16. Sousa RV, Clementino MA, Gomes MC, Martins CC, Granville-Garcia AF, Paiva SM (2011) Malocclusion and quality of life in Brazilian preschoolers. Eur J Oral Sci **122**: 223-229.
17. Thelen DS, Bardsen A, Astrom AN (2011) Applicability of an Albanian version of the OIDP in an adolescent population. Int J Paediatr Dent **21**: 289-298.
18. Feu D, de Oliveira BH, de Oliveira Almeida MA, Kiyak HA, Miguel JA (2010) Oral health-related quality of life and orthodontic treatment seeking. Am J Orthod Dentofacial Orthop**138**: 152-159.
19. Ghijselings I, Brosens V, Willems G, Fieuws S, Clijmans M, Lemiere J. (2014) Normative and self-perceived orthodontic treatment need in 11- to 16-year-old children. Eur J Orthod **36**: 179-185.
20. Kragt L, Tiemeier H, Wolvius EB, Ongkosuwito EM (2015) Measuring oral health-related quality of life in orthodontic patients with a short version of the childrens oral health impact profile. Journal of Public Health Dent [Epub ahead of print]
21. Zhang M, McGrath C, Hagg U (2009) Orthodontic treatment need and oral health-related quality among children. Community Dent Health **26**: 58-61.
22. Montiel-Company JM, Bellot-Arcis C, Almerich-Silla JM (2013) Validation of the psychosocial impact of dental aesthetics questionnaire (pidaq) in spanish adolescents. Med Oral Patol Oral Cir Bucal 18: e168-e173.
23. Abanto J, Carvalho TS, Mendes FM, Wanderley MT, Bönecker M, Raggio DP (2011) Impact of oral diseases and disorders on oral health-related quality of life of preschool children. Community Dent Oral Epidemiol **39**: 105-114.
24. Aguilar-Diaz FC, Irigoyen-Camacho ME, Borges-Yanez SA 2011 Oral-health-related quality of life in schoolchildren in an endemic fluorosis area of Mexico. Qual Life Res **20**: 1699-1706.
25. Anosike AN, Sanu OO, da Costa OO (2010) Malocclusion and its impact on quality of life of school children in Nigeria. West Afr J Med **29**: 417-424.
26. Asgari I, Ahmady AE, Yadegarfar G, Eslamipour F (2013) Evaluation of orthodontic treatment need by patient-based methods compared with normative method. Dent Res J (Isfahan) **10**: 636-642.
27. Barbosa TS, Tureli MCM, Gaviao MBD (2009) Validity and reliability of the Child Perceptions Questionnaires applied in Brazilian children. Bmc Oral Health **9**: 13.
28. Bhayat A, Ali MAM (2014) Validity and reliability of the Arabic short version of the child oral health-related quality of life questionnaire (CPQ 11-14) in Medina, Saudi Arabia. East Mediterr Health J **20**: 409-414.

29. Bekes K, John MT, Zyriax R, Schaller HG, Hirsch C (2012) The German version of the Child Perceptions Questionnaire (CPQ-G11-14): Translation process, reliability, and validity in the general population. Clin Oral Invest **16**: 165-171.
30. Bernabe E, de Oliveira CM, Sheiham A (2008) Comparison of the discriminative ability of a generic and a condition-specific OHRQoL measure in adolescents with and without normative need for orthodontic treatment. Health Qual Life Outcomes **6**: 64.
31. Bernabe E, Sheiham A, de Oliveira CM (2008) Condition-specific impacts on quality of life attributed to malocclusion by adolescents with normal occlusion and class I, II and III malocclusion. Angle Orthod **78**: 977-982.
32. Bernabe E, de Oliveira CM, Sheiham A, Tsakos G (2009) Comparison of the Generic and Condition-Specific Forms of the Oral Impacts on Daily Performances (OIDP) Index. J Publ Health Dent **69**: 176-181.
33. Bernabe E, Krisdapong S, Sheiham A, Tsakos G (2009) Comparison of the discriminative ability of the generic and condition-specific forms of the Child-OIDP index: A study on children with different types of normative dental treatment needs. Community Dent Oral Epidemiol **37**: 155-162.
34. Brown A, Al-Khayal Z (2006) Validity and reliability of the Arabic translation of the child oral-health-related quality of life questionnaire (CPQ11-14) in Saudi Arabia. Int J Paediatr Dent **16**: 405-411.
35. Carvalho AC, Paiva SM, Viegas CM, Scarpelli AC, Ferreira FM, Pordeus IA (2013) Impact of malocclusion on oral health-related quality of life among Brazilian preschool children: a population-based study. Braz Dent J **24**: 655-661.
36. Dawoodbhoy I, Delgado-Angulo EK, Bernabe E (2013) Impact of malocclusion on the quality of life of Saudi children. Angle Orthod **83**: 1043-1048.
37. Foster Page LA, Thomson WM, Jokovic A, Locker D (2005) Validation of the Child Perceptions Questionnaire (CPQ 11-14). J Dent Res **84**: 649-652.
38. Foster Page LA, Thomson WM, Ukra A, Farella M (2013) Factors influencing adolescents' oral health-related quality of life (OHRQoL). Int J Paediatr Dent **23**: 415-423.
39. Gomes MC, Pinto-Sarmento TC, Costa EM, Martins CC, Granville-Garcia AF, Paiva SM (2014) Impact of oral health conditions on the quality of life of preschool children and their families: A cross-sectional study. Health Qual Life Outcomes **12**: 55.
40. Herkrath FJ, Rebelo MA, Herkrath AP, Vettore MV (2013) Comparison of normative methods and the sociodental approach to assessing orthodontic treatment needs in 12-year-old shoolchildren. Oral Health Prev Dent 11: 211-220.
41. Hvaring CL, Birkeland K, Astrom AN (2014) Discriminative ability of the generic and condition specific Oral Impact on Daily Performance (OIDP) among adolescents with and without hypodontia. BMC Oral Health **14**: 57.
42. Kolawole KA, Otuyemi OD, Oluwadaisi AM (2011) Assessment of oral health-related quality of life in Nigerian children using the Child Perceptions Questionnaire (CPQ 11-14). Eur J Paediatr Dent **12**: 55-59.
43. Kramer PF, Feldens CA, Ferreira SH, Bervian J, Rodrigues PH, Peres MA (2013) Exploring the impact of oral diseases and disorders on quality of life of preschool children. Community Dent Oral Epidemiol **41**: 327-335.
44. Laing E, Cunningham SJ, Jones S, Moles D, Gill D. (2010) Psychosocial impact of hypodontia in children. Am J Orthod Dentofacial Orthop **137**: 35-41.
45. Locker D, Jokovic A, Tompson B, Prakash P (2007) Is the child perceptions questionnaire for 11-14 year olds sensitive to clinical and self-perceived variations in orthodontic status? Community Dent Oral Epidemiol **35**: 179-185.
46. Manjith CM, Karnam SK, Manglam S, Praveen MN, Mathur A (2012) Oral Health-Related Quality of Life (OHQoL) among adolescents seeking orthodontic treatment. J Contemp Dental Pract **13**: 294-298.
47. Marques LS, Ramos-Jorge ML, Paiva SM, Pordeus IA (2006) Malocclusion: Esthetic impact and quality of life among Brazilian schoolchildren. Am J Orthod Dentofacial Orthop **129**: 424-427.
48. Mbawalla HS, Mtaya M, Masalu JR, Brudvik P, Astrom AN (2011) Discriminative ability of the generic and condition-specific Child-Oral Impacts on Daily Performances (Child-OIDP) by the Limpopo-Arusha School Health (LASH) Project: A cross-sectional study. BMC Pediatr **11**: 45.
49. Onyeaso CO (2009) Orthodontic treatment complexity and need with associated oral health-related quality of life in Nigerian adolescents. Oral Health Prev Dent **7**: 235-241.
50. Paula JS, Leite IC, Almeida AB, Ambrosano GM, Pereira AC, Mialhe FL (2012) The influence of oral health conditions, socioeconomic status and home environment factors on schoolchildren's self-perception of quality of life. Health Qual Life Outcomes **10**: 6.
51. Paula DF, Silva ÉT, Campos AC, Nuñez MO, Leles CR (2011) Effect of anterior teeth display during smiling on the self-perceived impacts of malocclusion in adolescents. Angle Orthod **81**: 540-545.
52. Peres KG, Cascaes AM, Leão AT, Côrtes MI, Vettore MV (2013) Sociodemographic and clinical aspects of quality of life related to oral health in adolescents. Rev De Saude Publica, **47**: 19-28.
53. Sardenberg F, Martins MT, Bendo CB, Pordeus IA, Paiva SM, Auad SM, Vale MP (2013) Malocclusion and oral health-related quality of life in Brazilian school children. Angle Orthod **83**: 83-89.
54. Scapini A, Feldens CA, Ardenghi TM, Kramer PF (2013) Malocclusion impacts adolescents' oral health-related quality of life. Angle Orthod **83**: 512-518.
55. Scarpelli AC, Paiva SM, Viegas CM, Carvalho AC, Ferreira FM, Pordeus IA (2013) Oral health-related quality of life among Brazilian preschool children. Community Dent Oral Epidemiol **41**: 336-344.

56. Schuch HS Dos Santos Costa F, Torriani DD, Demarco FF, Goettems ML (2014) Oral health-related quality of life of schoolchildren: impact of clinical and psychosocial variables. Int J Paediatr Dent **25**: 358-365.
57. Ukra A, Foster Page LA, Thomson WM, Farella M, Tawse Smith A, Beck V (2013) Impact of malocclusion on quality of life among New Zealand adolescents. N Z Dent J **109**: 18-23.
